# Counteracting Colon Cancer by Inhibiting Mitochondrial Respiration and Glycolysis with a Selective PKCδ Activator

**DOI:** 10.3390/ijms24065710

**Published:** 2023-03-16

**Authors:** Cláudia Bessa, Joana B. Loureiro, Matilde Barros, Vera M. S. Isca, Vilma A. Sardão, Paulo J. Oliveira, Raquel L. Bernardino, Carina Herman-de-Sousa, Maria Adelina Costa, Paulo Correia-de-Sá, Marco G. Alves, Patrícia Rijo, Lucília Saraiva

**Affiliations:** 1 LAQV/REQUIMTE, Laboratόrio de Microbiologia, Departamento de Ciências Biolόgicas, Faculdade de Farmácia, Universidade do Porto, 4050-313 Porto, Portugal; 2 CBIOS—Research Center for Biosciences & Health Technologies, Universidade Lusófona de Humanidades e Tecnologias, 1749-024 Lisboa, Portugal; 3 Research Institute for Medicines (iMED.Ulisboa), Faculdade de Farmácia, Universidade de Lisboa, 1649-003 Lisboa, Portugal; 4 CNC—Center for Neuroscience and Cell Biology, CIBB—Centre for Innovative Biomedicine and Biotechnology, University of Coimbra, 3004-504 Coimbra, Portugal; 5 Multidisciplinary Institute of Aging (MIA-Portugal), University of Coimbra, 3004-504 Coimbra, Portugal; 6 Endocrine and Metabolic Research, UMIB—Unit for Multidisciplinary Research in Biomedicine, ICBAS—School of Medicine and Biomedical Sciences, University of Porto, 4050-313 Porto, Portugal; 7 Laboratory for Integrative and Translational Research in Population Health (ITR), University of Porto, 4200-465 Porto, Portugal; 8 Laboratório de Farmacologia e Neurobiologia, Center for Drug Discovery and Innovative Medicines (MedInUP), Instituto de Ciências Biomédicas Abel Salazar, Universidade do Porto (ICBAS-UP), 4050-313 Porto, Portugal; 9 Departamento de Química, Instituto de Ciências Biomédicas Abel Salazar, Universidade do Porto (ICBAS-UP), 4050-313 Porto, Portugal; 10 Laboratory of Physiology, Department of Immuno-Physiology and Pharmacology, ICBAS—School of Medicine and Biomedical Sciences, University of Porto, 4050-313 Porto, Portugal

**Keywords:** Roy-Bz, PKCδ, anticancer agent, OXPHOS, glycolysis

## Abstract

Metabolic reprogramming is a central hub in tumor development and progression. Therefore, several efforts have been developed to find improved therapeutic approaches targeting cancer cell metabolism. Recently, we identified the 7*α*-acetoxy-6*β*-benzoyloxy-12-*O*-benzoylroyleanone (Roy-Bz) as a PKCδ-selective activator with potent anti-proliferative activity in colon cancer by stimulating a PKCδ-dependent mitochondrial apoptotic pathway. Herein, we investigated whether the antitumor activity of Roy-Bz, in colon cancer, could be related to glucose metabolism interference. The results showed that Roy-Bz decreased the mitochondrial respiration in human colon HCT116 cancer cells, by reducing electron transfer chain complexes I/III. Consistently, this effect was associated with downregulation of the mitochondrial markers cytochrome *c* oxidase subunit 4 (COX4), voltage-dependent anion channel (VDAC) and mitochondrial import receptor subunit TOM20 homolog (TOM20), and upregulation of synthesis of cytochrome *c* oxidase 2 (SCO2). Roy-Bz also dropped glycolysis, decreasing the expression of critical glycolytic markers directly implicated in glucose metabolism such as glucose transporter 1 (GLUT1), hexokinase 2 (HK2) and monocarboxylate transporter 4 (MCT4), and increasing *TP53*-induced glycolysis and apoptosis regulator (TIGAR) protein levels. These results were further corroborated in tumor xenografts of colon cancer. Altogether, using a PKCδ-selective activator, this work evidenced a potential dual role of PKCδ in tumor cell metabolism, resulting from the inhibition of both mitochondrial respiration and glycolysis. Additionally, it reinforces the antitumor therapeutic potential of Roy-Bz in colon cancer by targeting glucose metabolism.

## 1. Introduction

Despite the huge investment in research and care, the increasing cancer incidence and mortality remains a major public health concern worldwide [1]. The anticancer therapies available so far are often ineffective mainly due to the acquisition of drug resistance and disease recurrence [2]. As such, efforts have been made to search for more reliable targets for cancer treatment. The metabolic reprogramming observed in cancer cells is driven by genetic mutations in addition to stressful and fluctuating microenvironment conditions to sustain their high proliferation rates [3]. This observation is characterized by a dramatic increase in glucose uptake and lactate production, instead of pyruvate, even in aerobic conditions [4]. In fact, unlike non-tumor cells, where adenosine triphosphate (ATP) is preferentially produced by oxidative phosphorylation (OXPHOS) in mitochondria, tumor cells largely depend on glycolysis for energy production, a phenomenon commonly referred to as the ‘Warburg effect’. Remarkably, this metabolic switch confers several advantages to cancer cells, particularly by providing intermediates for different biosynthetic pathways to meet the demands for rapidly dividing cells, and an adaptation to hypoxic conditions often observed in solid tumors [5]. These alterations were recognized as distinctive features of cancer cell bioenergetics and extensively explored as therapeutic targets in cancer treatment. However, in contrast to what Warburg originally hypothesized, the respiration of all cancer cells is not compromised [6,7,8], and tumor cell metabolism functions as an integrative system shared between glycolysis and OXPHOS, which allows cancer cells to adapt to specific conditions according to the location within the tumor microenvironment [9]. Based on these findings, mitochondrial metabolism has received significant attention as a target for the development of new anticancer drugs. Therefore, considering the tumor cell metabolic heterogeneity, the combined use of inhibitors of glycolysis and mitochondrial respiration has represented an attractive strategy to eradicate the tumor and overcome therapeutic resistance.

Protein kinase C (PKC) is a family of serine/threonine kinases that critically regulates several signaling transduction pathways involved in key cellular processes, such as cell survival, proliferation, apoptosis, metastasis, and metabolism [10,11,12,13]. PKC isozymes can be grouped based on their primary structure and activation cofactors into classical (cPKCs: α, βI/II, γ), novel (nPKCs: δ, ε, η, θ) and atypical (aPKCs: ζ, ι/λ) sub-families. In particular, dysregulation of PKCδ, an isozyme ubiquitously expressed in mammalian tissues, has been widely implicated in the onset of several metabolic disorders [14,15]. However, the interference of PKCδ in glucose metabolism of cancer cells still needs to be further elucidated.

**Figure 1:**
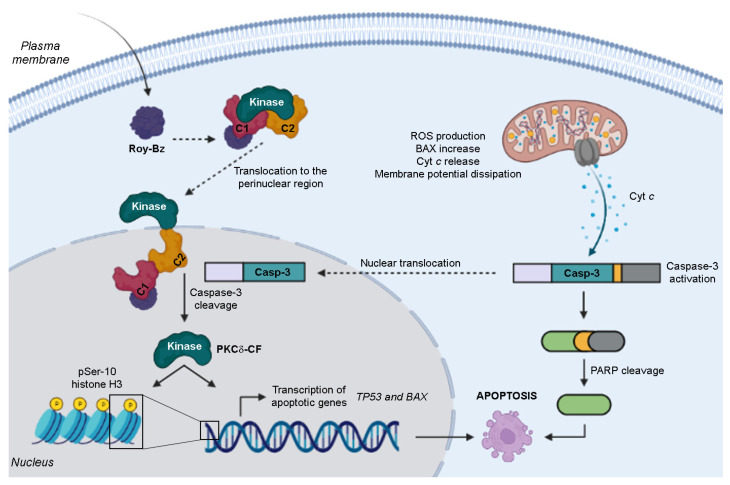
Schematic representation of the molecular mechanism of action of Roy-Bz-induced apoptosis. Roy-Bz binds to the PKCδ-C1 domain, inducing its translocation to the perinuclear region. In the nucleus, PKCδ is cleaved by caspase-3, resulting in the generation of the PKCδ catalytic fragment (PKCδ-CF), which in turns phosphorylates nuclear substrates, such as histone H3 on Ser-10, that promotes apoptosis. Additionally, it triggers the transcription of apoptotic genes, namely *TP53* and *BAX*, thus promoting a mitochondrial apoptotic pathway, with subsequent cytochrome *c* (cyt *c*) release, activation of caspase-3, PARP cleavage, and ultimately apoptosis induction.

Recent data from our group demonstrated that 7*α*-acetoxy-6*β*-benzoyloxy-12-*O*-benzoylroyleanone (Roy-Bz) is the first PKCδ-selective activator with promising antitumor properties in colon cancer [16]. Roy-Bz selectively activates PKCδ by establishing direct hydrogen bonds with *Gly253*, *Thr242* and *Gln257* in the C1-domain, which stabilize the molecule from both sides of the cleft [16]. Roy-Bz inhibits the proliferation of colon cancer cells by triggering a PKCδ-dependent mitochondrial apoptotic pathway involving caspase-3 activation (Figure 1). Notably, the PKCδ-dependent anticancer activity was recapitulated in vivo using xenograft mouse models of control and PKCδ-knockdown human colon cancer cells [16]. To further understand the molecular mechanism underlying the reported antitumor activity of Roy-Bz, herein, we investigated the impact of Roy-Bz on glucose metabolism of colon cancer cells.

## 2. Results

### 2.1. Roy-Bz Modulates Mitochondrial Activity Decreasing Cellular Respiration in Colon Cancer Cells

**Figure 2:**
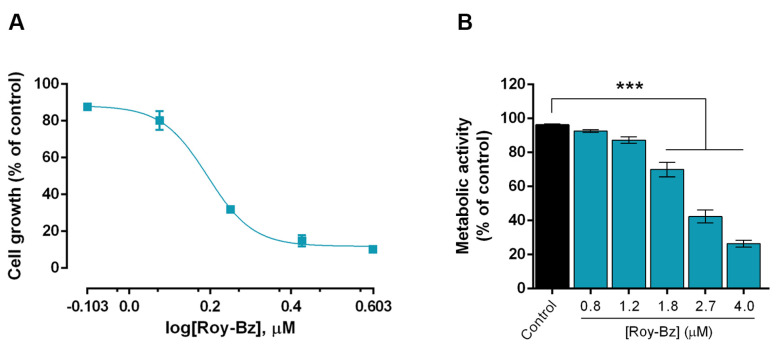
Roy-Bz has cytotoxic effect on colon cancer cells by decreasing their metabolic activity. (**A**,**B**) HCT116 cells were treated with increasing concentrations (0.8 to 4.0 μM) of Roy-Bz or vehicle (DMSO), for 48 h. In (**A**), the dose-response curve for the growth of HCT116 cells was obtained from the SRB assay (IC_50_ value of 1.65 ± 0.15 μM). Data are mean ± SEM of four independent experiments; growth obtained with vehicle was set as 100%. In (**B**), metabolic activity was determined by resorufin fluorescence measurement after 360 min incubation, using resazurin assay. Data are expressed as percentage of vehicle and are mean ± SEM of three independent experiments. Statistical analysis was carried out with a one-way ANOVA test followed by a Dunnett’s post hoc test; *** significantly different from control (cells treated with vehicle) with *p* < 0.001.

As previously described by our group, the antiproliferative activity of Roy-Bz in colon cancer cells was associated with the activation of a PKCδ-dependent mitochondrial apoptotic pathway, inducing mitochondrial reactive oxygen species (ROS) production, mitochondrial membrane potential (∆ψ_m_) dissipation, and cytochrome *c* (cyt *c*) release to the cytosol [16]. Based on this, in the present work, we aimed to investigate whether Roy-Bz could also interfere with mitochondrial activity. For that, we first evaluated the cytotoxic effect of Roy-Bz on both cell mass and metabolic activity of colon HCT116 cells, using the sulforhodamine B (SRB) and resazurin reduction assays, respectively. Additionally, cells were incubated in low glucose medium to promote the ATP production mostly through OXPHOS pathway and to detect direct effects of Roy-Bz on OXPHOS. Under these conditions, Roy-Bz caused a concentration-dependent antiproliferative effect on HCT116 cells, with an IC_50_ value of 1.65 ± 0.15 μM (Figure 2A), which was higher than that reported for cells tested in standard medium (IC_50_ of 0.58 ± 0.05 μM) [16]. Likewise, Roy-Bz concentration-dependently decreased the metabolic activity/viability of HCT116 cells (Figure 2B).

**Figure 3:**
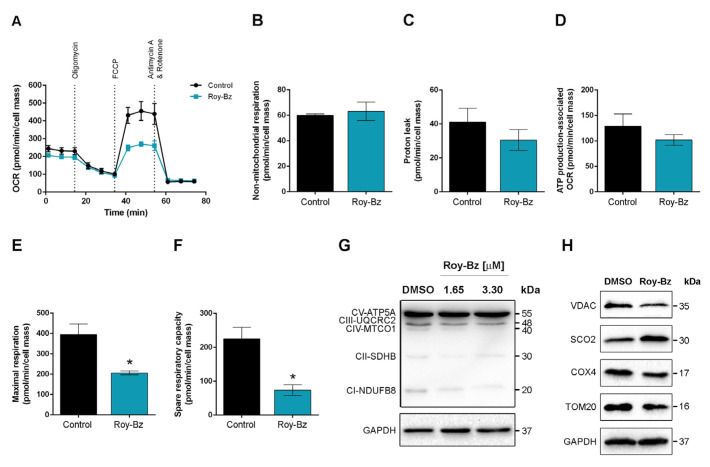
Roy-Bz inhibits cellular respiration in colon cancer cells. HCT116 cells were previously treated with 1.65 μM Roy-Bz or vehicle (control), except in (**G**) where they were treated with 1.65 and 3.30 μM Roy-Bz, for 48 h. (**A**) OCR were assessed with a Mito Stress Test using a Seahorse XF96 Analyzer, under basal conditions followed by sequential administration of 1 μM oligomycin, 0.5 μM FCCP, and 1 μM rotenone/1 μM antimycin A mix during the measurement. (**B**–**F**) Bioenergetics parameters, including (**B**) non-mitochondrial respiration (minimum rate measurement after rotenone/antimycin A injection), (**C**) proton leak associated OCR ((minimum rate measurement after oligomycin addition)-(non-mitochondrial respiration)), (**D**) ATP production associated OCR ((last rate measurement before oligomycin injection)-(minimum rate measurement after oligomycin injection)), (**E**) maximal respiration ((maximum rate measurement after FCCP injection)-(non-mitochondrial respiration)), (**F**) spare respiratory capacity ((maximal respiration)-(basal respiration)). Data are mean ± SEM of at least 4 independent experiments; values significantly different from control (* *p* < 0.05), unpaired Student’s *t*-test. (**G**,**H**) Expression levels of specific subunits of mitochondrial complexes I–V and mitochondrial-related proteins, respectively. Immunoblots represent one of the three independent experiments; glyceraldehyde-3-phosphate dehydrogenase (GAPDH) was used as a loading control.

We next investigated the effect of Roy-Bz on mitochondrial respiration using a Seahorse Bioanalyzer to measure the cellular oxygen consumption rates (OCR), a surrogate readout of mitochondrial function. As shown in Figure 3A, Roy-Bz treatment per se did not significantly alter the basal respiration of HCT116 cells. Moreover, different parameters associated with mitochondrial respiration were assessed in the real-time mode in cells treated with Roy-Bz, through sequential administration of electron transfer chain (ETC) modulators (Figure 3A). Oligomycin (complex V/ATP synthase inhibitor) was used to evaluate the OCR associated with mitochondrial ATP production, while the uncoupler carbonyl cyanide-4-(trifluoromethoxy) phenylhydrazone (FCCP) to measure the maximal respiratory capacity. A combination of rotenone (complex I inhibitor) and antimycin A (complex III inhibitor) was used to prevent the electron transport through ETC and to determine the cellular OCR that is not associated with mitochondria (non-mitochondrial respiration) [17,18]. Despite we observed no differences in some mitochondrial bioenergetic parameters, including non-mitochondrial respiration, OCR-linked to ATP production and OCR-linked to proton leak, Roy-Bz markedly decreased the maximal respiration and spare respiratory capacity of these cells, when compared to control non-treated cells (Figure 3B–F).

The Western blot analysis was also performed, using a total OXPHOS antibody cocktail that recognizes the epitopes of the specific subunits of mitochondrial complexes, in protein extracts of HCT116 cells, to establish a causal connection between the bioenergetic alterations and the amount of protein complexes I–V. Consistently with the analysis of mitochondrial activity, we observed a reduction in the protein levels of complexes I (NDUFB8 subunit) and III (UQCRC2 subunit), but not of complex V (ATP5A subunit; whose levels did not change even for the highest concentration tested, corresponding to twice the IC_50_), in HCT116 cells treated with Roy-Bz (Figure 3G).

The amount of major proteins involved in OXPHOS, including synthesis of cytochrome c oxidase 2 (SCO2) and cytochrome c oxidase subunit 4 (COX4), as well as of mitochondrial markers such as voltage-dependent anion-selective channel (VDAC) and mitochondrial import receptor subunit TOM20 homolog (TOM20), were also measured. The results revealed that Roy-Bz markedly reduced the protein levels of COX4, VDAC and TOM20, while it increased the SCO2 protein levels (Figure 3H).

**Figure 4:**
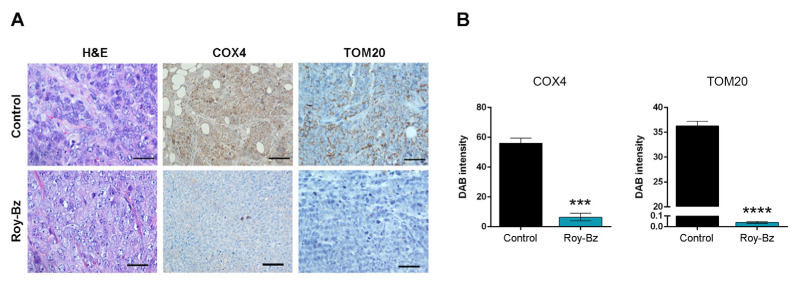
Roy-Bz inhibits cellular respiration in tumor tissues of human colon xenograft mouse models. (**A**) Representative images of IHC of mitochondrial markers (COX4 and TOM20) detected in tumor tissues of HCT116 xenografts treated with 10 mg/kg Roy-Bz or vehicle (control) and collected at the end of the treatment (scale bar = 5 μm; magnification = ×200; hematoxylin and eosin (H&E)). (**B**) Quantification of IHC staining of HCT116 xenograft tumor tissues was assessed by evaluation of DAB intensity. Data are mean ± SEM; values significantly different from control (*** *p* < 0.001, **** *p* < 0.0001), unpaired Student’s *t*-test.

The effect of Roy-Bz on cellular respiration was further evaluated in tumor xenografts resulting from the implantation of HCT116 cells in mice, which were obtained from our previous work [16]. As expected, a pronounced reduction of COX4 and TOM20 protein levels was also observed in tumor xenograft samples treated with Roy-Bz (Figure 4A,B).

### 2.2. Roy-Bz Inhibits Glycolysis of Colon Cancer Cells

**Figure 5:**
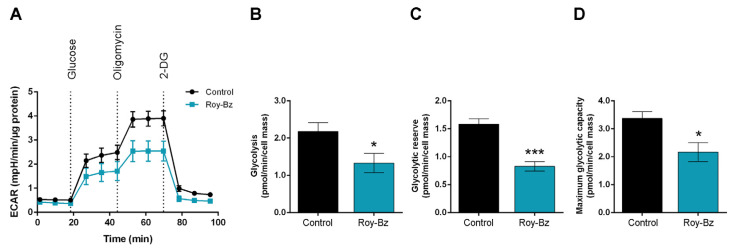
Roy-Bz inhibits the glycolytic pathway capacity in colon cancer cells. HCT116 cells were treated with 0.5 μM Roy-Bz or vehicle (DMSO), for 48 h. (**A**) ECAR curves were assessed with a Glycolysis Stress Test using a Seahorse XF24 Analyzer, under basal conditions followed by sequential injection of 10 mM glucose, 1 µM oligomycin, and 50 mM 2-DG during the measurement. (**B**–**D**) Bioenergetic parameters, including (**B**) glycolysis (maximal measurement after the addition of saturating amounts of glucose minus the measurement after adding 2-DG), (**C**) glycolytic reserve (maximal measurement following the addition of oligomycin minus maximal measurement before adding oligomycin), and (**D**) maximum glycolytic capacity (maximal measurement following the addition of oligomycin minus measurement after adding 2-DG). Data are mean ± SEM of at least 4 independent experiments; values significantly different from control (* *p* < 0.05, *** *p* < 0.001), unpaired Student’s *t*-test.

The ability of Roy-Bz to affect the glycolytic rate of HCT116 cells was explored by measuring the extracellular acidification rate (ECAR) by Seahorse analysis. For that, key glycolytic parameters were determined through sequential compound applications during the experiment, including glucose (glycolysis substrate), oligomycin (ATP synthase inhibitor; inhibits mitochondrial ATP production and shifts the energy metabolism to glycolysis), and 2-deoxyglucose (2-DG; glucose analogue that inhibits glycolysis through competitive binding to hexokinase 2 (HK2)). The results showed that Roy-Bz decreased ECAR after glucose injection (Figure 5A), thus indicating a lower overall glycolytic activity of Roy-Bz-treated cells, when compared to control. Although Roy-Bz did not interfere with the basal levels of extracellular acidification of cells (Figure 5A), it significantly reduced ECAR associated with glycolysis measured after glucose supplementation (Figure 5B). Moreover, it markedly dropped the glycolytic reserve, which is used as a measure of the capacity of the cells to switch their metabolism to glycolysis to compensate for OXPHOS defects (Figure 5C). Accordingly, Roy-Bz also downregulated the glycolytic capacity, which is directly proportional to the maximal capacity of the cells to respond to a higher ATP demand (Figure 5D).

**Figure 6:**
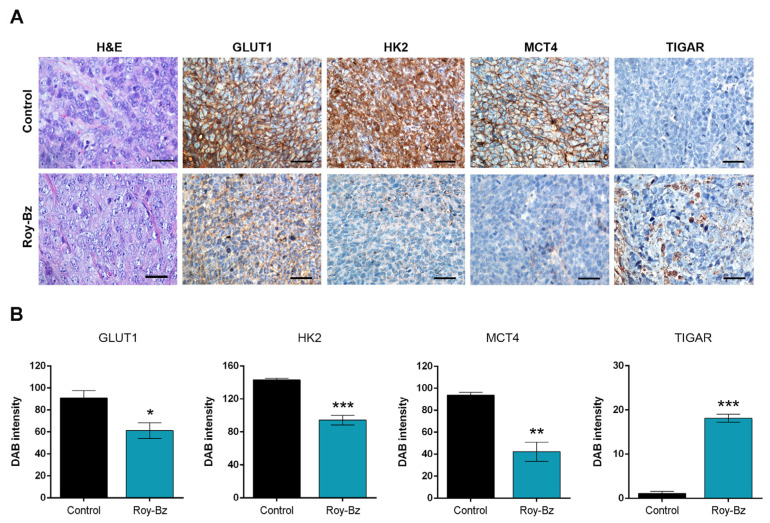
Roy-Bz reduces the levels of proteins involved in glycolysis in tumor tissues of human colon xenograft mouse models. (**A**) Representative images of IHC of glycolytic markers (GLUT1, HK2, MCT4, and TIGAR) detected in tumor tissues of HCT116 xenografts treated with 10 mg/kg Roy-Bz or vehicle (control) and collected at the end of the treatment (scale bar =5 μm; magnification = ×400; H&E). (**B**) Quantification of immunohistochemistry staining of HCT116 xenograft tumor tissues was assessed by evaluation of DAB intensity. Data are mean ± SEM; values significantly different from control (* *p* < 0.05, ** *p* < 0.01, *** *p* < 0.001), unpaired Student’s *t*-test.

The in vivo anti-glycolytic potential of Roy-Bz was also evaluated in tumor xenografts resulting from the implantation of HCT116 cells in mice, which were obtained from our previous work [16]. To this end, we assessed the protein levels of some glycolytic cell markers by immunohistochemistry (Figure 6A). In keeping with the ability to inhibit glycolysis, treatment with Roy-Bz significantly reduced the amount of glucose transporter 1 (GLUT1), HK2, and monocarboxylate transporter 4 (MCT4) proteins, while increasing the levels of TP53-induced glycolysis and apoptosis regulator (TIGAR) (Figure 6A,B). Altogether, these results strengthen the anti-glycolytic properties of Roy-Bz.

## 3. Discussion

Metabolic reprogramming is a recognized hallmark of malignant transformation [3], and it has become the subject of intensive research for the design of new therapeutic approaches. The strong dependence of tumors on glycolysis led to the development of a huge number of glycolytic enzyme inhibitors, including natural compounds [19,20,21]. However, recent advances have demonstrated that many tumors rely on OXPHOS to meet the energetic demands, thus setting the focus on mitochondrial metabolism [9,22]. Although glycolysis is a key pathway to support tumor growth, mitochondria also largely support the bioenergetic and biosynthetic needs of growing cancer cells [23,24,25,26]. Interestingly, the biguanides, metformin and phenformin, initially approved by the Food and Drug Administration (FDA) agency for the treatment of type-2 diabetes [27], are two of the main drugs used to inhibit ETC complex I, therefore depleting mitochondrial respiration in cancer cells. Remarkably, metformin has been tested in several clinical trials for cancer patients, having shown encouraging results in a Phase III clinical trial designed to evaluate its efficacy in treating of early-stage breast cancer (NCT01101438) [28,29].

In the present work, it is shown that the potent antitumor activity of Roy-Bz in colon cancer previously demonstrated [16] may also be related to its dual ability to inhibit mitochondrial respiration and glycolysis, inducing the downregulation of enzymes directly implicated in the glycolytic pathway of colon cancer. The results herein obtained support that Roy-Bz impairs OXPHOS in colon cells through a decrease of the protein levels of specific subunits of ETC complexes I/III, resulting in the reduction of the mitochondrial respiration. Interestingly, this is in line with the reported inhibition of complex I by metformin and xanthohumol [30,31]. As a PKCδ-selective activator [16], this decrease of mitochondrial respiration by Roy-Bz strongly implicates an involvement of PKCδ. In fact, multiple determinants unique of PKCδ have been identified that drive an isozyme-specific interaction with mitochondria outer membrane, thus enabling the regulation of cellular metabolism [32].

In a previous work, we reported that Roy-Bz significantly increased mitochondrial oxidative stress and led to ∆ψ_m_ dissipation [16] (Figure 1), which are events implicated in the induction of mitochondrial-apoptotic cell death upon complex I inhibition in pancreatic, lung, and cervix cancer cells [31,33]. Additionally, the release of pro-apoptotic factors such as cyt *c* to the cytosol, which in turn triggers the activation of caspases such as the executioner caspase-3, are also involved in apoptosis stimulation [34]. In fact, this is in agreement with the molecular mechanism of action described for Roy-Bz-induced apoptosis in colon cancer [16]. Interestingly, some studies have indicated that p53-mediated-upregulation of SCO2 stimulates OXPHOS, thereby increasing ROS and leading to cell death [35]. However, in this study, the increased protein levels of SCO2 are associated with an inhibition of mitochondrial respiration, which may indicate that SCO2 by itself is not enough to ensure the normal functionality of the respiratory chain. In opposition, all the other markers analyzed, including COX4, VDAC and TOM20, were downregulated, thus supporting the inhibition of the mitochondrial activity in colon cells upon Roy-Bz treatment.

Besides the effect on cellular respiration, our in vitro results indicate that Roy-Bz also downregulates glycolysis in colon cells, negatively affecting critical parameters of glycolytic flux. Further corroborating these results, in vivo, Roy-Bz regulated the expression of some enzymes directly implicated in the glycolytic pathway, namely reducing the levels of GLUT1 (involved in the initial uptake of glucose into the cell [36]), HK2 (that catalyzes the first committed step of the glycolytic flux with phosphorylation of glucose to glucose-6-phosphate [37]), and MCT4 (that mediates the export of lactate [38]). Conversely, it upregulated TIGAR, which is a negative regulator of the glycolytic flux through inhibition of phosphofructose kinase 1 (PFK1) activity by degrading its allosteric activator fructose-2,6-bisphosphate [39,40,41].

There are some studies that have contributed to the elucidation of the function of PKCδ in glucose metabolism, under non-tumor conditions. In fact, in a recent work, data from metabolomic analysis demonstrated an inhibition of glycolysis and tricarboxylic acid cycle (TCA), which was associated with an upregulation of other metabolic pathways in PKCδ-deficient mice [42]. Additionally, regarding mitochondrial respiration, it was described that PKCδ is implicated in the stimulation of the pyruvate dehydrogenase complex activity, thereby increasing the flux of fuel entering the TCA cycle by converting pyruvate to acetyl CoA in the mitochondrial matrix [43,44,45,46]. Despite this, the role of PKCδ in glucose metabolism of tumor cells still needs to be clarified. Conversely to that observed in a non-tumor context, using a PKCδ-selective activator, our work evidenced an inhibitory effect of PKCδ on mitochondrial respiration and glycolysis of colon cancer cells. Therefore, this dual role of PKCδ in tumor cell metabolism highlights this PKC isozyme as a promising therapeutic target in cancer.

Overall, this work provides insights into the role of PKCδ in tumor cell metabolism. Moreover, it strongly supports the ability of Roy-Bz to target both ATP producing pathways of glucose metabolism in cancer cells, thereby reinforcing its potential as an anticancer agent. These data also pave the way to the development of new therapeutic strategies targeting cellular metabolism with PKC activators. Finally, the exploitation of Roy-Bz as a dual inhibitor of glycolysis and OXPHOS in combination therapy with other metabolic drugs or chemotherapeutic agents may represent a promising approach in colon cancer therapy.

## 4. Materials and Methods

### 4.1. Compounds

7*α*-Acetoxy-6*β*-benzoyloxy-12-*O*-benzoylroyleanone (Roy-Bz) was obtained by semi-synthesis from the natural diterpenoid 7α-acetoxy-6β-hydroxyroyleanone isolated from *Plectranthus grandidentatus* Gürke plant, following procedures formerly described in ref. [47]. The compound was dissolved in dimethyl sulfoxide (DMSO; Merck Life Science, Algés, Portugal).

### 4.2. Human Cancer Cell Lines and Culture Conditions

The human colon adenocarcinoma HCT116 cell line was provided by B. Vogelstein (The Johns Hopkins Kimmel Cancer Center, Baltimore, MD, USA), and was routinely cultured in RPMI 1640 medium with UltraGlutamine from Lonza (VWR, Carnaxide, Portugal) supplemented with 10% fetal bovine serum (FBS) from Gibco (Alfagene, Lisboa, Portugal), here designated as the standard medium. For the experiments described in Section 4.3, Section 4.4 and Section 4.5 of this section, cells were cultured in Dulbecco′s Modified Eagle′s Medium (DMEM) glucose and phenol red-free (D5030; Merck Life Science, Algés, Portugal), supplemented with 5 mM glucose (Merck Life Science, Algés, Portugal), 4 mM L-glutamine (Merck Life Science, Algés, Portugal), 1 mM sodium pyruvate (Merck Life Science, Algés, Portugal), 21 mM sodium bicarbonate (Merck Life Science, Algés, Portugal), and supplemented with 10% FBS (pH 7.4), designated as low glucose medium. The cell line was maintained at 37 °C in a humidified atmosphere of 5% CO_2_, and routinely tested for mycoplasma infection by using the MycoAlert^TM^ PLUS mycoplasma detection kit (Lonza).

### 4.3. Cell Proliferation Assay

Cellular proliferation was determined using the sulforhodamine B (SRB) assay, which measures cell mass. Briefly, HCT116 cells were seeded in 96-well plates, at a final density of 4.5 × 10^3^ cells/well, for 24 h, and then treated with serial dilutions of Roy-Bz (from 0.8 to 4.0 μM) or vehicle (DMSO only) for 48 h, in normoxia conditions (95% O_2_). Thereafter, cells were fixed with 10% trichloroacetic acid (TCA; Panreac, Frilabo, Maia, Portugal), stained with 0.4% SRB (Merck Life Science, Algés, Portugal), and washed with 1% acetic acid (VWR). The bound dye was solubilized in 10 mM Tris Base (Merck Life Science, Algés, Portugal), and the absorbance was measured at 510 nm in a microplate reader (Biotek Instruments Inc., Winsooki, VT, USA). The maximum concentration of solvent used in this assay (0.25% DMSO) was included as control, and the values obtained were set as 100%. Half-maximal inhibitory concentration (IC_50_; concentration that causes 50% of growth inhibition) value was determined from the concentration-response curve.

### 4.4. Cellular Metabolic Viability Assay

The cytotoxic effect of Roy-Bz was also assessed by measuring the metabolic activity using the resazurin reduction assay. For that, HCT116 cells were seeded in 96-well plates, at a final density of 4.5 × 10^3^ cells/well, for 24 h, and then treated with serial dilutions of Roy-Bz (from 0.8 to 4.0 μM) or vehicle for 48 h, in normoxia conditions. After treatment, the medium was replaced by a fresh medium containing 10 μg/mL resazurin prepared in sterile phosphate buffer saline (1X) (PBS; Merck Life Science, Algés, Portugal) and left to react for 30 min as described [48]. Metabolic activity was determined based on the reduction of resazurin to resorufin by the dehydrogenases present in viable cells, and subsequent resorufin fluorescence measurement at 540/590 nm (ex/em) in a microplate reader at 30, 60, 240 and 360 min, and expressed in relative fluorescence units (rfu’s). The maximum concentration of solvent used in this assay (0.25% DMSO) was included as a control, and the values obtained were set as 100%. Data were expressed as a percentage of control.

### 4.5. Cellular Oxygen Consumption Measurements

The OCR and ECAR of HCT116 cells were analyzed by the Agilent Seahorse XFe96 Analyzer (Agilent Technologies, Inc., Santa Clara, CA, USA), using the Agilent Seahorse XF Cell Mito Stress Test Kit (Agilent Technologies, Soquímica, Lisboa, Portugal), according to the manufacturer’s instructions. Briefly, HCT116 cells were seeded in Seahorse XF96 cell culture microplate, at a final density of 2.5 × 10^4^ cells/well in D5030 medium with 5 mM glucose, 4 mM L-glutamine, 1 mM sodium pyruvate, 21 mM sodium bicarbonate, and supplemented with 10% FBS (pH 7.4), for 24 h. Thereafter, cells were treated with 1.65 μM Roy-Bz (IC_50_ value in low glucose conditions), for 48 h in normoxia conditions. XF96 sensor cartridge for each cell plate was placed in a 96-well calibration plate containing 200 μL/well calibration buffer and left to hydrate overnight at 37 °C. After treatments, the culture medium was removed, replaced by pre-warmed XF Cell Mito Stress Test Assay medium, consisting of D5030 with 5 mM glucose, 4 mM L-glutamine, 1 mM sodium pyruvate, and without FBS and sodium bicarbonate (adjusted pH to 7.4 with 1N NaOH), and cells were incubated at 37 °C for 1 h. The compounds (oligomycin, FCCP, and the mix of rotenone and antimycin A) provided by the Seahorse XF Cell Mito Stress Test Kit were reconstituted with the previously prepared assay medium. For OCR and ECAR measurements, XF96 sensor cartridge was pre-loaded with oligomycin (8 μM) into reagent delivery port A, FCPP (4.5 μM) into port B, and rotenone/antimycin A mix (10 μM of each) into reagent delivery port C. Oligomycin, FCCP, and the mix of rotenone and antimycin A, were then pneumatically injected by the XF96 Analyzer into each well, performing a final concentration of 1 μM, 0.5 μM and 1 μM respectively. OCR and ECAR were measured for 12 cycles composed by 3 min mix and 5 min measurement. OCR and ECAR data were normalized by protein content, after cell lysis with RIPA Buffer (ChemicalNor, Valongo, Portugal) and protein quantification with BCA protein assay (Thermo Fisher, Porto Salvo, Portugal). As such, figures represent parameters calculated using normalized percentages or protein-adjusted values. Each condition was analyzed in duplicates and all calculations were made using the Seahorse Analytics platform.

### 4.6. Evaluation of the Glycolytic Pathway Capacity after Glucose Starvation

The glycolytic pathway capacity after glucose starvation was analyzed by measuring the extracellular acidification rate using the Agilent Seahorse XFe24 Analyzer (Agilent Technologies, Inc., Santa Clara, CA, USA), with the Agilent Seahorse XF Glycolysis Stress Test Kit (Agilent Technologies, Soquímica, Lisboa, Portugal), according to the manufacturer’s instructions. Briefly, HCT116 cells were seeded in Seahorse XF24 cell culture microplates, at a final density of 2.0 × 10^4^ cells/well in a standard medium, for 24 h. Then, cells were treated with 0.5 μM Roy-Bz for 48 h in normoxia conditions. After the incubation period, the culture medium was exchanged to assay medium (XF base medium DMEM supplemented with 2 mM glutamine and 1 mM Pyruvate) (Agilent Technologies, Cedar Creek, TX, USA), and cells were incubated at 37 °C for 40 min. The compounds (glucose, oligomycin, 2-DG) provided by the Seahorse XF Glycolysis Stress Test Kit were reconstituted with the previously prepared assay medium. For ECAR measurements, the sequential injection of 10 mM glucose, 1 μM oligomycin and 50 mM 2-DG was performed by the XF24 Analyzer, with 3 measurements of ECAR between each injection. Seahorse data were normalized to protein content, after cell lysis with RIPA Buffer (ChemicalNor, Valongo, Portugal) and protein quantification with BCA protein assay (Thermo Fisher, Porto Salvo, Portugal). As such, figures represent parameters calculated using normalized percentages or protein-adjusted values. Each condition was analyzed in duplicates and all calculations were made using the Seahorse Analytics platform.

### 4.7. Western Blot Analysis

HCT116 cells were seeded in 6-well plates at a final density of 1.5 × 10^5^ cells/well for 24 h, and thereafter treated with different concentrations of Roy-Bz for 48 h. Upon treatment, cells were lysed and total protein extracts were quantified using the Bradford reagent (Merck Life Science, Algés, Portugal). Proteins were then run in SDS-PAGE and transferred to a Whatman nitrocellulose membrane from Protan (VWR, Carnaxide, Portugal). After blocking, proteins were identified using specific primary antibodies followed by HRP-conjugated secondary antibodies described in Supplementary Materials (Table S1). The signal was detected with the ECL Amersham kit from GE Healthcare (VWR, Carnaxide, Portugal) and band intensities were quantified as described in [49]. Whole-blot images are provided in Supplementary Materials.

### 4.8. Immunohistochemistry Analysis

Tumor tissues from colon HCT116 cancer xenografts treated with 10 mg/kg Roy-Bz or vehicle (DMSO only) applied by intraperitoneal injection twice a week, for a total of five administrations, were obtained from previous study [16]. The tissues were fixed in 10% formalin, embedded in paraffin, sectioned at 4 μm, and stained with hematoxylin and eosin (H&E) or for immunohistochemistry, as described in [50]. Briefly, antigen retrieval was performed by boiling the sections for 20 min in citrate (pH 6.0) or EDTA (pH 8.0) buffer. The antibodies used are listed in Supplementary Table S1. Immunostaining documentation was performed using the UltraVision Quanto Detection System HRP DAB Kit, from Lab Vision Thermo Scientific (Grupo Taper SA, Sintra, Portugal), according to the manufacturer’s instructions. Evaluation of 3,30-diaminobenzidine (DAB) intensity and quantification of marked cells were performed using the Image J software (Laboratory for optical and computational instrumentation, University of Wisconsin-Madison, Madison, WI, USA). Images were obtained at 400× magnification using an Eclipse E400 fluorescence microscope (Nikon, Tokyo, Japan) equipped with a Digital Sight camera system (Nikon DS-5Mc) and the software Nikon ACT-2U (Izasa Carnaxide, Portugal).

### 4.9. Statistical Analysis

Data are mean ± SEM values of at least three independent experiments. The two-tailed unpaired Student’s *t*-test was used for the comparison of two sets of data, and the one-way ANOVA test followed by the Dunnett’s multiple comparisons post hoc test to compare groups with one independent variable; *p* < 0.05 was considered statistically significant. The GraphPad Prism version 6.0 (San Diego, CA, USA) software was used for graphs and statistical analyses.

## Data Availability

The data presented in this study are available on request from the corresponding authors.

## Supplementary Materials

The following supporting information can be downloaded at: https://www.mdpi.com/article/10.3390/ijms24065710/s1.

## References

[1] Bray F., Laversanne M., Weiderpass E., Soerjomataram I. (2021). The ever-increasing importance of cancer as a leading cause of premature death worldwide. Cancer.

[2] Snyder V., Reed-Newman T.C., Arnold L., Thomas S.M., Anant S. (2018). Cancer Stem Cell Metabolism and Potential Therapeutic Targets. Front. Oncol..

[3] Hanahan D., Weinberg R.A. (2011). Hallmarks of cancer: The next generation. Cell.

[4] Warburg O. (1956). On the origin of cancer cells. Science.

[5] Liberti M.V., Locasale J.W. (2016). Correction to: “The Warburg Effect: How Does it Benefit Cancer Cells?”: [Trends in Biochemical Sciences, 41 (2016) 211]. Trends Biochem. Sci..

[6] Chance B., Castor L.N. (1952). Some Patterns of the Respiratory Pigments of Ascites Tumors of Mice. Science.

[7] Weinhouse S. (1956). On respiratory impairment in cancer cells. Science.

[8] Weinhouse S. (1967). Hepatomas. Science.

[9] Cassim S., Vucetic M., Zdralevic M., Pouyssegur J. (2020). Warburg and Beyond: The Power of Mitochondrial Metabolism to Collaborate or Replace Fermentative Glycolysis in Cancer. Cancers.

[10] Griner E.M., Kazanietz M.G. (2007). Protein kinase C and other diacylglycerol effectors in cancer. Nat. Rev. Cancer.

[11] Steinberg S.F. (2008). Structural basis of protein kinase C isoform function. Physiol. Rev..

[12] Reyland M.E. (2009). Protein kinase C isoforms: Multi-functional regulators of cell life and death. Front. Biosci. Landmark Ed..

[13] Newton A.C. (2010). Protein kinase C: Poised to signal. Am. J. Physiol. Endocrinol. Metab..

[14] Lien C.F., Chen S.J., Tsai M.C., Lin C.S. (2021). Potential Role of Protein Kinase C in the Pathophysiology of Diabetes-Associated Atherosclerosis. Front. Pharmacol..

[15] Sparks R., Lui A., Bader D., Patel R., Murr M., Guida W., Fratti R., Patel N.A. (2019). A specific small-molecule inhibitor of protein kinase CdeltaI activity improves metabolic dysfunction in human adipocytes from obese individuals. J. Biol. Chem..

[16] Bessa C., Soares J., Raimundo L., Loureiro J.B., Gomes C., Reis F., Soares M.L., Santos D., Dureja C., Chaudhuri S.R. (2018). Discovery of a small-molecule protein kinase Cdelta-selective activator with promising application in colon cancer therapy. Cell Death Dis..

[17] Little A.C., Kovalenko I., Goo L.E., Hong H.S., Kerk S.A., Yates J.A., Purohit V., Lombard D.B., Merajver S.D., Lyssiotis C.A. (2020). High-content fluorescence imaging with the metabolic flux assay reveals insights into mitochondrial properties and functions. Commun. Biol..

[18] Lajqi T., Marx C., Hudalla H., Haas F., Grosse S., Wang Z.Q., Heller R., Bauer M., Wetzker R., Bauer R. (2021). The Role of the Pathogen Dose and PI3Kgamma in Immunometabolic Reprogramming of Microglia for Innate Immune Memory. Int. J. Mol. Sci..

[19] Abdel-Wahab A.F., Mahmoud W., Al-Harizy R.M. (2019). Targeting glucose metabolism to suppress cancer progression: Prospective of anti-glycolytic cancer therapy. Pharmacol. Res..

[20] Zhang Y., Li Q., Huang Z., Li B., Nice E.C., Huang C., Wei L., Zou B. (2022). Targeting Glucose Metabolism Enzymes in Cancer Treatment: Current and Emerging Strategies. Cancers.

[21] Zhao Y., Chard Dunmall L.S., Cheng Z., Wang Y., Si L. (2022). Natural products targeting glycolysis in cancer. Front. Pharmacol..

[22] Sainero-Alcolado L., Liano-Pons J., Ruiz-Perez M.V., Arsenian-Henriksson M. (2022). Targeting mitochondrial metabolism for precision medicine in cancer. Cell Death Differ..

[23] Koppenol W.H., Bounds P.L., Dang C.V. (2011). Otto Warburg’s contributions to current concepts of cancer metabolism. Nat. Rev. Cancer.

[24] Goto M., Miwa H., Shikami M., Tsunekawa-Imai N., Suganuma K., Mizuno S., Takahashi M., Mizutani M., Hanamura I., Nitta M. (2014). Importance of glutamine metabolism in leukemia cells by energy production through TCA cycle and by redox homeostasis. Cancer Investig..

[25] Goto M., Miwa H., Suganuma K., Tsunekawa-Imai N., Shikami M., Mizutani M., Mizuno S., Hanamura I., Nitta M. (2014). Adaptation of leukemia cells to hypoxic condition through switching the energy metabolism or avoiding the oxidative stress. BMC Cancer.

[26] Viale A., Pettazzoni P., Lyssiotis C.A., Ying H., Sanchez N., Marchesini M., Carugo A., Green T., Seth S., Giuliani V. (2014). Oncogene ablation-resistant pancreatic cancer cells depend on mitochondrial function. Nature.

[27] Suissa S., Azoulay L. (2014). Metformin and cancer: Mounting evidence against an association. Diabetes Care.

[28] Saraei P., Asadi I., Kakar M.A., Moradi-Kor N. (2019). The beneficial effects of metformin on cancer prevention and therapy: A comprehensive review of recent advances. Cancer Manag. Res..

[29] Coyle C., Cafferty F.H., Vale C., Langley R.E. (2016). Metformin as an adjuvant treatment for cancer: A systematic review and meta-analysis. Ann. Oncol..

[30] Mogavero A., Maiorana M.V., Zanutto S., Varinelli L., Bozzi F., Belfiore A., Volpi C.C., Gloghini A., Pierotti M.A., Gariboldi M. (2017). Metformin transiently inhibits colorectal cancer cell proliferation as a result of either AMPK activation or increased ROS production. Sci. Rep..

[31] Zhang B., Chu W., Wei P., Liu Y., Wei T. (2015). Xanthohumol induces generation of reactive oxygen species and triggers apoptosis through inhibition of mitochondrial electron transfer chain complex I. Free Radic. Biol. Med..

[32] Wu-Zhang A.X., Murphy A.N., Bachman M., Newton A.C. (2012). Isozyme-specific interaction of protein kinase Cdelta with mitochondria dissected using live cell fluorescence imaging. J. Biol. Chem..

[33] Palorini R., Simonetto T., Cirulli C., Chiaradonna F. (2013). Mitochondrial complex I inhibitors and forced oxidative phosphorylation synergize in inducing cancer cell death. Int. J. Cell Biol..

[34] Tait S.W., Green D.R. (2010). Mitochondria and cell death: Outer membrane permeabilization and beyond. Nat. Rev. Mol. Cell Biol..

[35] Madan E., Gogna R., Kuppusamy P., Bhatt M., Mahdi A.A., Pati U. (2013). SCO2 induces p53-mediated apoptosis by Thr845 phosphorylation of ASK-1 and dissociation of the ASK-1-Trx complex. Mol. Cell Biol..

[36] Marger M.D., Saier M.H. (1993). A major superfamily of transmembrane facilitators that catalyse uniport, symport and antiport. Trends Biochem. Sci..

[37] Wang L., Xiong H., Wu F., Zhang Y., Wang J., Zhao L., Guo X., Chang L.J., Zhang Y., You M.J. (2014). Hexokinase 2-mediated Warburg effect is required for PTEN- and p53-deficiency-driven prostate cancer growth. Cell Rep..

[38] Halestrap A.P., Meredith D. (2004). The SLC16 gene family-from monocarboxylate transporters (MCTs) to aromatic amino acid transporters and beyond. Pflugers Arch..

[39] Bensaad K., Cheung E.C., Vousden K.H. (2009). Modulation of intracellular ROS levels by TIGAR controls autophagy. EMBO J..

[40] Berkers C.R., Maddocks O.D., Cheung E.C., Mor I., Vousden K.H. (2013). Metabolic regulation by p53 family members. Cell Metab..

[41] Li H., Jogl G. (2009). Structural and biochemical studies of TIGAR (TP53-induced glycolysis and apoptosis regulator). J. Biol. Chem..

[42] Loots D.T., Adeniji A.A., Van Reenen M., Ozturk M., Brombacher F., Parihar S.P. (2022). The metabolomics of a protein kinase C delta (PKCdelta) knock-out mouse model. Metabolomics.

[43] Caruso M., Maitan M.A., Bifulco G., Miele C., Vigliotta G., Oriente F., Formisano P., Beguinot F. (2001). Activation and mitochondrial translocation of protein kinase Cdelta are necessary for insulin stimulation of pyruvate dehydrogenase complex activity in muscle and liver cells. J. Biol. Chem..

[44] Acin-Perez R., Hoyos B., Gong J., Vinogradov V., Fischman D.A., Leitges M., Borhan B., Starkov A., Manfredi G., Hammerling U. (2010). Regulation of intermediary metabolism by the PKCdelta signalosome in mitochondria. FASEB J..

[45] Acin-Perez R., Hoyos B., Zhao F., Vinogradov V., Fischman D.A., Harris R.A., Leitges M., Wongsiriroj N., Blaner W.S., Manfredi G. (2010). Control of oxidative phosphorylation by vitamin A illuminates a fundamental role in mitochondrial energy homoeostasis. FASEB J..

[46] Gong J., Hoyos B., Acin-Perez R., Vinogradov V., Shabrova E., Zhao F., Leitges M., Fischman D., Manfredi G., Hammerling U. (2012). Two protein kinase C isoforms, delta and epsilon, regulate energy homeostasis in mitochondria by transmitting opposing signals to the pyruvate dehydrogenase complex. FASEB J..

[47] Rijo P., Simoes M.F., Francisco A.P., Rojas R., Gilman R.H., Vaisberg A.J., Rodriguez B., Moiteiro C. (2010). Antimycobacterial metabolites from Plectranthus: Royleanone derivatives against Mycobacterium tuberculosis strains. Chem. Biodivers..

[48] Silva F.S.G., Starostina I.G., Ivanova V.V., Rizvanov A.A., Oliveira P.J., Pereira S.P. (2016). Determination of Metabolic Viability and Cell Mass Using a Tandem Resazurin/Sulforhodamine B Assay. Curr. Protoc. Toxicol..

[49] Raimundo L., Espadinha M., Soares J., Loureiro J.B., Alves M.G., Santos M.M.M., Saraiva L. (2018). Improving anticancer activity towards colon cancer cells with a new p53-activating agent. Br. J. Pharmacol..

[50] Raimundo L., Paterna A., Calheiros J., Ribeiro J., Cardoso D.S.P., Piga I., Neto S.J., Hegan D., Glazer P.M., Indraccolo S. (2021). BBIT20 inhibits homologous DNA repair with disruption of the BRCA1-BARD1 interaction in breast and ovarian cancer. Br. J. Pharmacol..

