# Supplementary material for: Counteracting Colon Cancer by Inhibiting Mitochondrial Respiration and Glycolysis with a Selective PKCδ Activator
Source: Int J Mol Sci. 2023 Mar 16;24(6):5710. doi: 10.3390/ijms24065710 (PMC10054007; doi:10.3390/ijms24065710)

International Journal of *Molecular Sciences*

MDPI

Supplementary materials

Counteracting colon cancer by inhibiting mitochondrial respiration and glycolysis with a selective PKCδ activator

Cláudia Bessa[1], Joana B. Loureiro[1], Matilde Barros[1], Vera M. S. Isca[2,3], Vilma A. Sardão[4,5], Paulo J. Oliveira[4], Raquel L. Bernardino[6,7], Carina Herman-de-Sousa[8], Maria Adelina Costa[8,9], Paulo Correia-de-Sá[8], Marco G. Alves[6,7,10], Patrícia Rijo[2,3,*], Lucília Saraiva[1,*]

[1] LAQV/REQUIMTE, Laboratório de Microbiologia, Departamento de Ciências Biológicas, Faculdade de Farmácia, Universidade do Porto, 4050-313 Porto, Portugal
[2] CBIOS-Research Center for Biosciences & Health Technologies, Universidade Lusófona de Humanidades e Tecnologias, 1749-024 Lisboa, Portugal
[3] Research Institute for Medicines (iMED.Ulisboa), Faculdade de Farmácia, Universidade de Lisboa, 1649-003 Lisboa, Portugal
[4] CNC-Center for Neuroscience and Cell Biology, CIBB - Centre for Innovative Biomedicine and Biotechnology, University of Coimbra, 3004-504 Coimbra, Portugal
[5] Multidisciplinary Institute of Aging (MIA-Portugal), University of Coimbra, 3004-504 Coimbra, Portugal
[6] Endocrine and Metabolic Research, UMIB - Unit for Multidisciplinary Research in Biomedicine, ICBAS - School of Medicine and Biomedical Sciences, University of Porto, 4050-313 Porto, Portugal
[7] Laboratory for Integrative and Translational Research in Population Health (ITR), University of Porto, 4200-465 Porto, Portugal
[8] Laboratório de Farmacologia e Neurobiologia / Center for Drug Discovery and Innovative Medicines (MedInUP), Instituto de Ciências Biomédicas Abel Salazar - Universidade do Porto (ICBAS-UP), 4050-313, Porto, Portugal
[9] Departamento de Química, Instituto de Ciências Biomédicas Abel Salazar - Universidade do Porto (ICBAS-UP), 4050-313, Porto, Portugal
[10] Laboratory of Physiology, Department of Immuno-Physiology and Pharmacology, ICBAS - School of Medicine and Biomedical Sciences, University of Porto, 4050-313 Porto, Portugal
* Correspondence: lucilia.saraiva@ff.up.pt; patricia.rijo@ulusofona.pt

Table S1: List of antibodies used in the work.
OXPHOS, oxidative phosphorylation; WB, western blot; glucose transporter 1, GLUT1; immunohistochemistry, IHC;

**Antibodies/Applications**	**Supplier**	**Identifier**
	Primary antibodies	
Total OXPHOS antibody cocktail (Mouse monoclonal) (WB)	Abcam	Cat# ab110413; RRID:AB_2629281
GLUT1 (Rabbit polyclonal) (IHC)		Cat# ab652; RRID:AB_305540
HK2 (3D3) (Mouse monoclonal) (IHC)	Merck Millipore	Cat# MABN702
SCO2 (Rabbit polyclonal) (WB)	Proteintech	Cat# 21223-1-AP; RRID:AB_10694574
COX4 (F-8) (Mouse monoclonal) (WB/IHC)	Santa Cruz Biotechnology	Cat# sc-376731; RRID:AB_2904544
GAPDH (6C5) (Mouse monoclonal) (WB)		Cat# sc-32233; RRID: AB_627679
MCT4 (H-90) (Rabbit polyclonal) (IHC)		Cat# sc-50329; RRID:AB_2189333
VDAC (B-6) (mouse monoclonal) (WB)	Santa Cruz Biotechnology	Cat# sc-390996; RRID:AB_2750920
TOM20 (F-10) (Mouse monoclonal) (WB/IHC)	Santa Cruz Biotechnology	Cat# sc-17764; RRID:AB_628381
TIGAR (Rabbit polyclonal) (IHC)	Merck Millipore	Cat# AB10545; RRID:AB_10807181
	Secondary antibodies	
Anti-mouse horseradish-peroxidase (HRP)-conjugated (WB)	Santa Cruz Biotechnology	Cat# sc-2005; RRID: AB_631736
Anti-rabbit horseradish-peroxidase (HRP)-conjugated (WB)		Cat# sc-2004; RRID: AB_631746

hezokinase 2, HK2; synthesis of cytochrome *c* oxidase 2, SCO2; cytochrome c oxidase subunit 4, COX4; glyceraldehyde-3-phosphate dehydrogenase, GAPDH; monocarboxylate transporter 4, MCT4; voltage-dependent anion-selective channel, VDAC; mitochondrial import receptor subunit TOM20 homolog, TOM20; *TP53*-induced glycolysis and apoptosis regulator, TIGAR.

Figure S1: Whole blot images.

Figure 2G

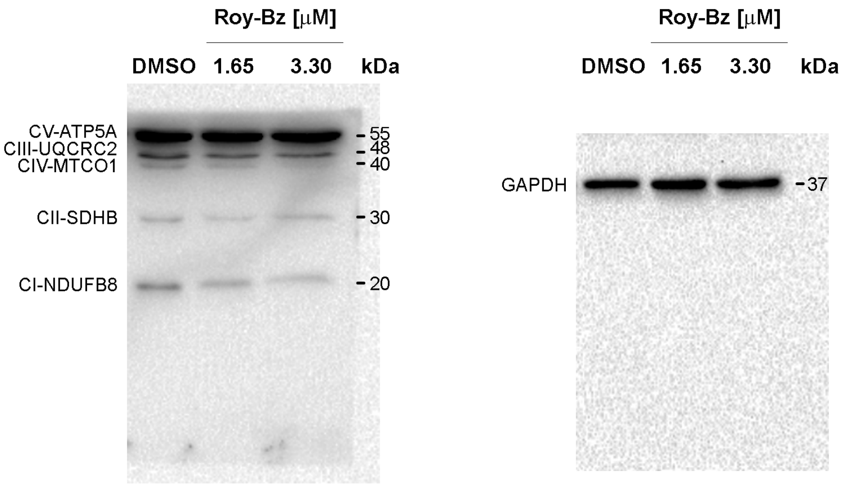

Figure 2H

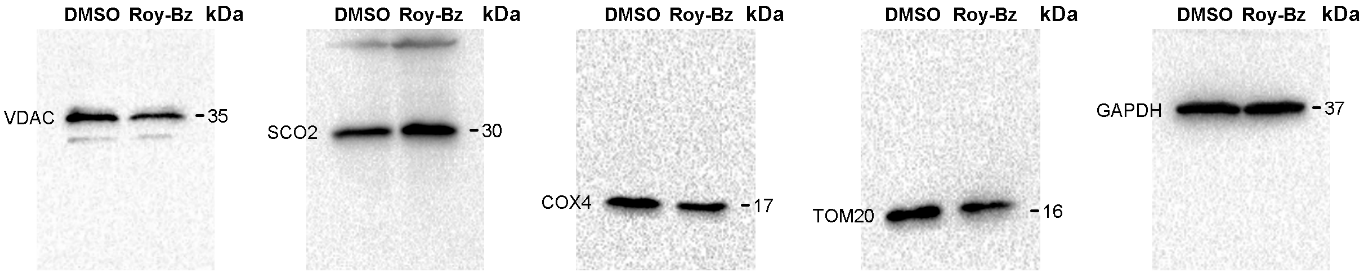

Supplement: Supplementary file 1 [file ijms-24-05710-s001.zip › ijms-2261598-supplementary.pdf]
